# Soil Bacterial Community Structure and Co-occurrence Pattern during Vegetation Restoration in Karst Rocky Desertification Area

**DOI:** 10.3389/fmicb.2017.02377

**Published:** 2017-12-01

**Authors:** Liang Xue, Huadong Ren, Sheng Li, Xiuhui Leng, Xiaohua Yao

**Affiliations:** ^1^Research Institute of Subtropical Forestry, Chinese Academy of Forestry, Fuyang, China; ^2^Observation and Research Station for Rock Desert Ecosystem, Puding, China

**Keywords:** bacterial communities, co-occurrence, vegetation restoration, karst, rocky desertification

## Abstract

Vegetation restoration has been widely used in karst rocky desertification (KRD) areas of southwestern China, but the response of microbial community to revegetation has not been well characterized. We investigated the diversity, structure, and co-occurrence patterns of bacterial communities in soils of five vegetation types (grassland, shrubbery, secondary forest, pure plantation and mixed plantation) in KRD area using high-throughput sequencing of the 16S rRNA gene. Bray-Curtis dissimilarity analysis revealed that 15 bacterial community samples were clustered into five groups that corresponded very well to the five vegetation types. Shannon diversity was positively correlated with pH and Ca^2+^ content but negatively correlated with organic carbon, total nitrogen, and soil moisture. Redundancy analysis indicated that soil pH, Ca^2+^ content, organic carbon, total nitrogen, and soil moisture jointly influenced bacterial community structure. Co-occurrence network analysis revealed non-random assembly patterns of bacterial composition in the soils. *Bryobacter*, GR-WP33-30, and *Rhizomicrobium* were identified as keystone genera in co-occurrence network. These results indicate that diverse soil physicochemical properties and potential interactions among taxa during vegetation restoration may jointly affect the bacterial community structure in KRD regions.

## Introduction

Southwestern China is one of the three distribution centers of Karst in the world. From the 1950s to the 1980s, Karst regions in China experienced severe soil erosion, vegetation loss, and desertification caused by intensive anthropogenic activity, referred to as karst rocky desertification (KRD; Wang et al., 2004; Jiang et al., 2014). Revegetation has been widely implemented in ecological conservation and restoration efforts because it improves soil nutrient conditions and other environmental factors (Crouzeilles et al., 2016; Feng et al., 2016). In recent decades, vegetation restoration has been used to overcome the ecological degradation in these regions (Qi et al., 2013). For example, a number of ecological projects have been implemented in southwestern China, such as the Yangtze River Shelter-Forest Project, the Pearl River Shelter-Forest Project and diversified water and soil conservation projects, all of which have been based on vegetation restoration (Wang et al., 2004; Jiang et al., 2014).

Soil microbial communities play an essential role in shaping the aboveground biodiversity and functioning of terrestrial ecosystems by driving biogeochemical processes and mediating nutrient turnover (Doran and Zeiss, 2000; Bardgett and van der Putten, 2014). Thus, microbial community structure is an essential indicator of the health and sustainability of an ecosystem (Harris, 2003; Zak et al., 2003; Lewis et al., 2010). Co-occurrence network analysis provides insight into the structure of microbial communities and the interactions among microorganisms (Barberán et al., 2012). However, soil microbial communities and co-occurrence patterns to vegetation restoration are largely unknown. This information provides invaluable reference to the appropriate management and conservation of the degraded karst ecosystem.

Previous studies showed that microbial community structure can be affected by soil properties such as pH, C, N and moisture (Yergeau et al., 2007; Lauber et al., 2009; Chong et al., 2010; Chu et al., 2011; Yu et al., 2012; Bakker et al., 2013; Schlatter et al., 2015), and plant factor such as vegetation type (Oh et al., 2012; Shi et al., 2015). In this study, we elucidate the relationships among vegetation type, soil physicochemical properties, and microbial community structure in the KRD area using high-throughput sequencing of the 16S rRNA gene. The main objectives of this study are to test the following hypotheses: (i) the bacterial communities differ among the principal vegetation types of the KRD areas, and (ii) vegetation-associated soil properties play significant impacts on bacterial community structure. Considering the critical role of microbial interactions in determining the soil microbial communities, co-occurrence patterns among bacterial communities were also explored in the KRD soils.

## Materials and Methods

### Study Area and Sampling

A field experiment was carried out in five types of vegetation at the San-do-qing Forestry Station (25°02′30″–25°58′22″N, E103°58′37″–104°49′48″), located in Fuyuan County, Yunnan Province. Fuyuan County is on the eastern Yunnan Karst plateau. This area has a northern subtropical monsoon climate. The mean annual temperature is 13.8°C, with a mean minimum temperature of 5.7°C in January and a mean maximum temperature of 19.8°C in July (Li et al., 2014). The main type of soil is red limestone. The KRD area covers 601.018 km^2^, accounting for 29.33% of the Karst region (Li et al., 2016). The five typical types of vegetation in the area are grassland, shrubbery, secondary forest, pure plantation, and mixed plantation. The grassland, shrubbery, and secondary forest are representative of three successional stages, having been abandoned as farmland 3, 12, and 45 years prior, respectively. The pure plantation and mixed plantation were afforested from grassland 25 years before sampling. The grassland is covered mainly by *Chrysopogon orientalis, Fragaria vesca*, and *Dicranopteris dichotoma*. The shrubbery is dominated by *Pyracantha fortuneana, Corylus yunnanensis*, and *Myrica nana*. The secondary forest is dominated by *Quercus aquifolioides* and *Lithocarpus dealbatus*. The pure plantation consists of *Pinus armandi*, whereas the mixed plantation is dominated by *Pinus yunnanensis* and *Alnus ferdinandi-coburgii*. The understory of all three types of forest is dominated by *Hypericum monogynum* and *Myrica nana*.

Three 20 × 20 m plots were established at each site in August 2015. The minimum distance between plots was 500 m to avoid pseudoreplication. Soil samples were collected from the surface soil (0–10 cm) with a stainless steel cutting ring (5 cm in diameter). Six soil cores were selected in an S-shaped pattern from each plot and mixed to form one replicate. All soils were transported to the laboratory immediately after collection in sterile plastic bags on dry ice and divided into two portions. One subset was stored at -80°C for DNA analysis, and the other subset was air dried for physicochemical analysis.

### Soil Physicochemical and Biological Parameters

Soil organic carbon (SOC) content was determined using potassium dichromate oxidation (Nelson and Sommers, 1982). Total nitrogen (TN) content was estimated with a TOC analyzer (Multi N/C 3100 TOC, Analytik, Jena, Germany). Soil moisture (SM) was analyzed by weighing the soil and calculating the mass lost after oven drying at 105°C until weight was stable (24 h). Soil pH was determined with a soil-to-water ratio of 1:2.5 (w/v) using a pH meter (FE20, Mettler-Toledo Instruments, China). Ca^2+^ content was measured using an atomic absorption spectrophotometer (ICE 3500, Thermo Scientific, United States).

### DNA Extraction and Purification

DNA was extracted directly from the soil samples using the Power Soil Extraction Kit (Mo Bio Laboratories, San Diego, CA, United States) according to the manufacturer’s instructions. The concentration and purity of the extracted DNA were measured using a Nanodrop 2000 spectrometer (Thermo Fisher Scientific, Wilmington, DE, United States). The soil DNA was stored at -20°C until use.

The V4–V5 variable regions of the bacterial 16S ribosomal RNA gene were amplified via polymerase chain reaction (PCR) using two universal eubacterial primer pairs, 515F (5′-GTGCCAGCMGCCGCGG-3′) and 907R (5′-CCGTCAATTCMTTTRAGTTT-3′; Xiong et al., 2012). The forward and reverse primers were tagged with adapter, pad, and linker sequences. Barcode sequences (10-mer) were added to the reverse primer to pool multiple samples into one run for sequencing. All primers were synthesized by Invitrogen Life Technologies (Shanghai, China). PCR amplification was conducted using TransGen AP221-02: TransStart Fastpfu DNA Polymerase (TransGen Biotech, Beijing, China) and performed in a GeneAmp 9700 thermal cycler (Applied Biosystems, Foster City, CA, United States). The reaction mixture included 4 μL 5× FastPfu buffer, 2 μL 2.5 mM dNTPs, 0.2 μL BSA, 0.8 μL each primer (5 μM), 10 ng template DNA, and H_2_O to a final volume of 20 μL. Thermal cycling conditions were as follows: 95°C for 3 min followed by 27 cycles of 95°C for 30 s, 55°C for 30 s, and 72°C for 45 s, with a final extension at 72°C for 10 min. All samples were amplified in triplicate. PCR amplification was detected using 2% agarose gel electrophoresis. The triplicate amplification products were pooled and purified using the AxyPrep DNA Gel Extraction Kit (AXYGEN, Union City, CA, United States) and then quantified using QuantiFluor^TM^-ST (Promega, United States).

### Bacterial 16S rRNA Gene Sequencing and Processing

The V4–V5 region (515F–907R) of the bacterial 16S rRNA gene was sequenced on the Illumina Miseq PE 250 platform. Bacterial raw reads were deposited in NCBI Sequence Read Archive (SRA) under the submission ID SUB2918840 and BioProject ID PRJNA397824. Processing of the 16S rRNA–derived sequence inventories was performed using QIIME (quantitative insights into microbial ecology; Caporaso et al., 2010). Briefly, partial 16S rRNA bacterial sequences were filtered using Mothur version 1.22.2 (Schloss et al., 2009) with the inclusion criteria of mean quality score ≥20 and length ≥ 250bp. Sequences were assigned to samples by exact matches of 10 bp barcodes. Then the Uchime algorithm was used to detect chimeric sequences from a chimera-free reference database (Edgar et al., 2011) via the Usearch tool. All chimeras were removed prior to further analysis. Operational taxonomic units (OTUs) were clustered at the 97% similarity level using UPARSE version 7.1 (Edgar, 2010)^1^. Final OTUs were generated based on the clustering results, and taxonomic assignment was performed with the RDP 16S Classifier^2^ (Wang et al., 2007).

### Statistical Analyses

Statistical analysis of OTU richness via Good’s coverage, Chao1, and Shannon’s index was performed with Mothur (version 1.22.2; Schloss et al., 2009). One-way analysis of variance (ANOVA) followed by Duncan’s multiple range test (DMRT) was performed to assess the significance of the effects of vegetation type on soil properties and microbial community composition and diversity using SPSS version 17.0 (SPSS Inc., Chicago, IL, United States). Bray-Curtis dissimilarity values were calculated to reveal the relationships among samples based on bacterial community composition. Shared and unique OTUs among the five vegetation types were used to generate a Venn diagram. The 50 most abundant OTUs among the five vegetation types were analyzed using the hierarchical clustering software Cluster version 3.0^3^ and visualized using Java TreeView version 1.1.6.^4^ Redundancy analysis (RDA) was performed with Canoco (version 4.5 for Windows; Ithaca, NY, United States) to determine which environmental variables best explained the assemblage’s variability. Forward selection was based on Monte Carlo permutation tests (permutations = 999). The ordination in the *x*- and *y*-axes and the length of the corresponding arrows indicated the importance of each physicochemical factor in explaining the taxon distribution across communities. The co-occurrence of OTUs in microbial communities across the five vegetation types was analyzed. To reduce network complexity and facilitate the identification of the core soil community, we selected OTUs with more than five sequences for further analysis (Barberán et al., 2012). Significant Spearman correlations (*p* < 0.01) were noted, and visualization of the co-occurrence network was conducted using the Fruchtermann-Feingold layout of the interactive platform Gephi version 0.9.0. Possible keystone genera were those that demonstrated high betweenness centrality values (Vick-Majors et al., 2014). The modular structure of the community was evaluated via the modularity index (Lambiotte et al., 2015).

## Results

### Soil Physicochemical Properties Associated with the Five Vegetation Types

Soil pH decreased as the vegetation developed from grassland (5.6) and shrubbery (5.6) to secondary forest (5.27), with pure plantation (4.69) exhibiting the lowest value (**Table 1**). In addition, no significant difference in Ca^2+^ concentration was observed among vegetation types except for pure plantation, which had the lowest value (0.37). In general, TN, SOC, and SM increased significantly (*p* < 0.05) as the vegetation changed from grassland to forest. Furthermore, carbon to nitrogen C:N ratios were comparable among the natural succession vegetation types (12.99–14.61), although pure plantation and mixed plantation showed higher values (14.9 and 15.79, respectively).

**Table 1:** Soil physicochemical properties of five vegetation types.

Physicochemical factor	Vegetation type				
	Grassland	Shrubbery	Secondary forest	Mixed plantation	Pure plantation
pH	5.60 ± 0.19a	5.60 ± 0.13a	5.27 ± 0.12b	4.80 ± 0.16c	4.69 ± 0.10c
Ca (g kg^-1^)	0.53 ± 0.02a	0.57 ± 0.04a	0.52 ± 0.03a	0.51 ± 0.04a	0.37 ± 0.03b
SM (%)	25.47 ± 1.61a	29.03 ± 1.30b	33.67 ± 1.38c	34.53 ± 0.59c	34.57 ± 0.55c
SOC (g kg^-1^)	24.91 ± 2.47a	25.03 ± 0.5a	28.27 ± 1.44b	32.8 ± 1.82c	31.07 ± 1.80bc
TN (g kg^-1^)	1.83 ± 0.03a	1.93 ± 0.04ab	1.94 ± 0.05b	2.05 ± 0.09c	2.09 ± 0.06c
C/N	13.63 ± 1.22a	12.99 ± 0.03ab	14.61 ± 1.12abc	15.97 ± 0.21c	14.90 ± 1.03bc

*Statistical significance was assessed by one-way ANOVA followed by DMRT, *P* < 0.05. Different lowercase letters showed statistically significant differences.*

### Distribution of Taxa and Phylotypes

We obtained a total of 1,182,391 high-quality bacterial V4–V5 Illumina sequences and 3778 OTUs (at 3% evolutionary distance) after applying all quality filters. Almost all sequences (99.95%) were between 350 and 400 bp, with an average read length of 396.21 bp. The number of sequences obtained from each sample ranged from 59,788 to 108,809. Good’s coverage values ranged from 98.9 to 99.4% (**Table 2**), with the number of OTUs increasing sharply before reaching a plateau, which indicates that the number of bacterial sequences obtained represented the bacterial communities well, as the rarefaction curves tended toward saturation (**Supplementary Figure S1**). The bacteria were from 32 phyla, 76 classes, 170 orders, 312 families, and 506 genera. The dominant phyla (except for Proteobacteria, which were characterized at the class level) across all samples were Acidobacteria (21.73–57.08%), Actinobacteria (2.21–22.89%), Alphaprotebacteria (9.95–16.8%), Chloroflexi (0.94–10.26%), Planctomycetes (3.59–9.79%), Deltaproteobacteria (4.21–8.64%), Gammaproteobacteria (3.34–7.34%), and Betaproteobacteria (1.42–3.97%) and to a lesser degree Gemmatimonadetes (0.063–6.02%), Bacteroidetes (0.88–3.38%), Armatimonadetes (0.26–1.81%), Nitrospirae (0–1.78%), and Latescibacteria (0–1.73%; >1%), which together accounted for more than 95% of bacterial sequences from each of the vegetation types (**Supplementary Figure S2**).

**Table 2:** Characteristics of soil bacteria richness and diversity indices under different vegetation types.

Sample ID	Vegetation type	Number of OTUs	Coverage (%)	Chao 1	Shannon’s index
A1	Grassland	2074	98.90	2447	6.16
A2	Grassland	2215	98.88	2589	6.24
A3	Grassland	2210	98.86	2573	6.24

**Average**		2166a	98.88a	2536a	6.21a

B1	Shrubbery	2057	98.94	2436	6.2
B2	Shrubbery	2065	99.01	2364	6.12
B3	Shrubbery	2106	99.94	2479	6.22

**Average**		2076a	99.30a	2426a	6.18a

C1	Secondary forest	1935	98.92	2345	5.92
C2	Secondary forest	1777	98.88	2233	5.48
C3	Secondary forest	1513	99.06	1876	5.48

**Average**		1741b	98.95a	2151b	5.56b

D1	Mixed plantation	1506	99.18	1796	5.57
D2	Mixed plantation	1567	99.07	1952	5.62
D3	Mixed plantation	1427	99.26	1690	5.56

**Average**		1500c	99.17a	1812c	5.58b

E1	Pure plantation	1022	99.31	1367	4.65
E2	Pure plantation	1058	99.43	1293	4.98
E3	Pure plantation	981	99.42	1224	4.52
**Average**		1020d	99.39a	1294d	4.72c

*Statistical significance was assessed by one-way ANOVA followed by DMRT, *P* < 0.05. Different lowercase letters showed statistically significant differences.*

### Bacterial Diversity and Differences in Community Structure among the Vegetation Types

According to OTU diversity estimated by Shannon’s index, the greatest bacterial diversity was in grassland and shrubbery soils (average = 6.21 and 6.18, respectively) followed by secondary forest and mixed plantation (average = 5.56 and 5.58, respectively), whereas pure plantation showed the lowest bacterial diversity (average = 4.72; **Table 2**). These results indicate that vegetation restoration plays an important role in determining soil bacterial diversity.

The relative abundance of each bacterial taxonomic group varied among the five vegetation types (**Supplementary Figure S2**). It is remarkable that the relative abundance of bacterial phyla associated with grassland and shrub differed significantly from the other three vegetation types. For example, the Acidobacteria and Gammaproteobacteria phylotypes were less abundant in grassland (33.95 and 3.49%, respectively) and shrubbery (21.80 and 3.56%) than in the secondary forest (49.53 and 5.57%), mixed plantation (50.20 and 5.55%), and pure plantation (53.62 and 6.82%) sites. Conversely, some taxa decreased markedly in relative abundance from grassland and shrubbery to the three forest types, including Actinobacteria, Betaproteobacteria, Gemmatimonadetes, and Nitrospirae.

### The Impact of Vegetation-Associated Soil Characteristics on Bacterial Community Composition and Diversity

**FIGURE 1:**
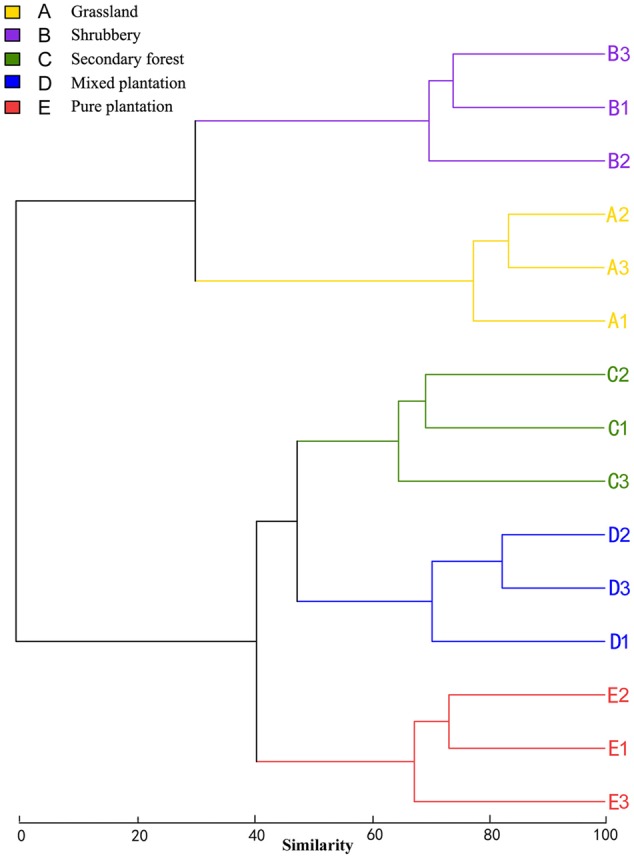
Clustering analysis of bacterial communities under five vegetation types based on OTU abundance-based Bray-Curtis similarity coefficients.

**FIGURE 2:**
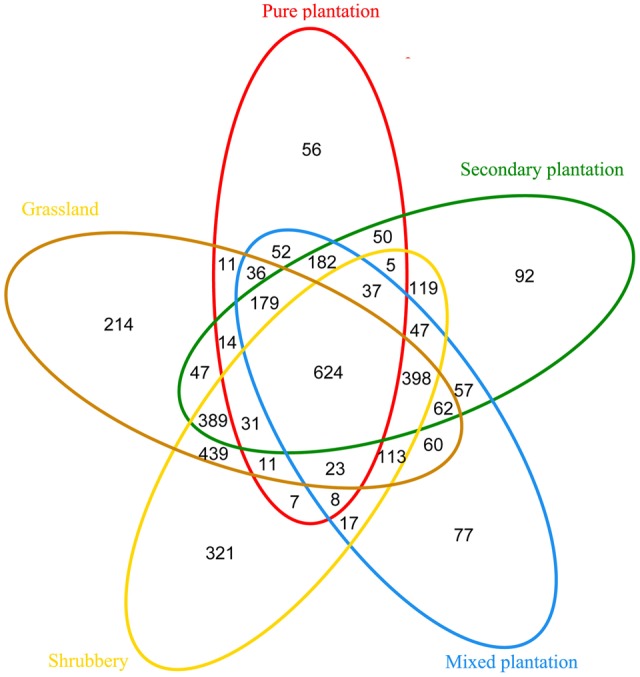
Venn diagram of exclusive and shared bacterial OTUs (at the 3% evolutionary distance) under five vegetation types.

Bray-Curtis dissimilarity analysis revealed that the 15 bacterial community samples clustered into five groups that corresponded very well to the five vegetation types (**Figure 1**). Clustering indicated that the grassland and shrubbery sites were closely related and secondary forest and mixed plantation shared a close relationship. A Venn diagram demonstrated that OTUs differed among the five vegetation types (**Figure 2**). The number of site-specific OTUs ranged from 56 (pure plantation) to 321 (shrubbery). In addition, a total of 624 OTUs were shared among all five vegetation types; these were defined as generalists. Generalist OTUs were composed of a number of bacterial groups, including Acidobacteria, Proteobacteria, Actinobacteria, and Bacteroidetes.

**FIGURE 3:**
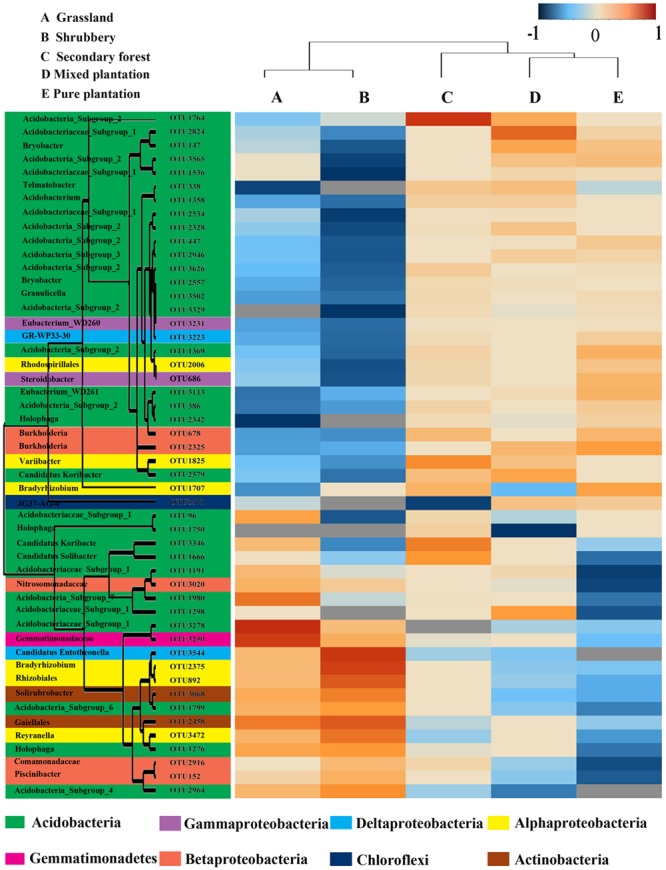
A heatmap diagram of the dominant 50 OTUs under five vegetation types.

To investigate the differences in the soil bacterial communities of the five vegetation types, we used heatmap analysis of the 50 most abundant OTUs, which highlighted their relative distributions and abundances (Supplementary Table S2). As shown in the heatmap (**Figure 3**), the abundance of these 50 dominant OTUs differed among the five vegetation types. The dominant OTUs in each vegetation type were also different. For example, the mixed plantation site was dominated by OTU2328 and OTU2824, whereas pure plantation was enriched by OTU686.

**FIGURE 4:**
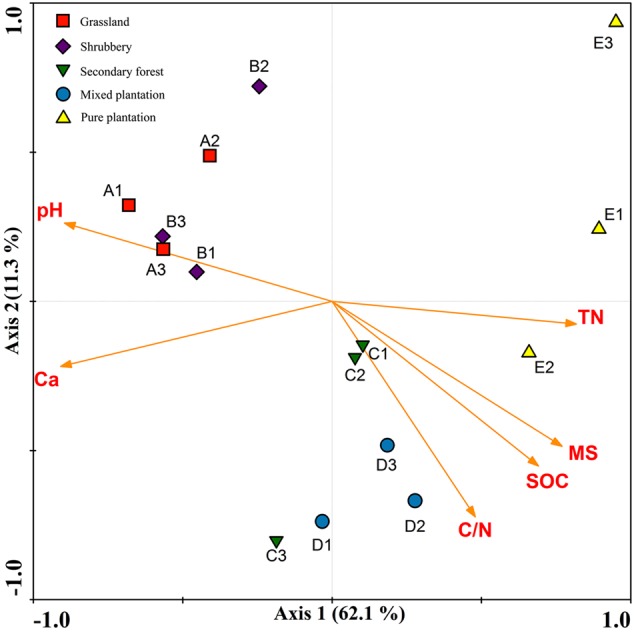
Redundancy analysis to show correlation between the bacterial communities and physicochemical properties under five vegetation types.

Redundancy analysis and a Monte Carlo permutation test were used to determine the relationships among six biochemical factors and bacterial community structure. All of the edaphic variables explained 81.2% of the variance, with axis 1 explaining 62.1% of the variance and axis 2 explaining another 11.3% (**Figure 4**). The major biochemical characteristics driving soil bacterial community composition were pH (*F* = 3.55, *p* = 0.001), soil moisture (*F* = 3.33, *p* = 0.001), Ca^2+^ (*F* = 2.90, *p* = 0.001), soil organic C (*F* = 2.724, *p* = 0.004), total N (*F* = 2.31, *p* = 0.004), and C/N (*F* = 2.051, *p* = 0.013).

### Bacterial Co-occurrence Network Analysis

**FIGURE 5:**
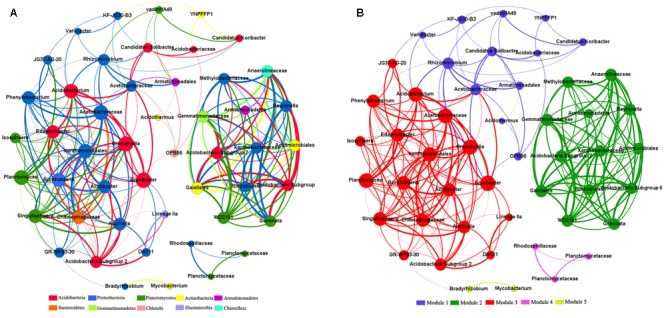
Co-occurring network of bacterial communities across five vegetation types based on correlation analysis. The nodes in network **(A)** are colored by phylum. The nodes in network **(B)** are colored by modularity class. The connections stands for a strong (spearman’s ρ > 0.6) and significant (*P* < 0.01) correlations. The size of each node is proportional to the relative abundance of specific genus. The thickness of each edge is proportional to the ρ.

Across all 15 samples from five Karst vegetation types, correlation network analysis showed 219 strong positive correlations among 49 genera (*R* > 0.6, *p* ≤ 0.01; **Figure 5**). The network of positive correlations formed three distinct major (≥10 OTUs) modules of co-occurring taxa. The average path length between two nodes (APL) was 2.03 edges with a diameter of six edges. The clustering coefficient (CC) was 0.72 and the modularity index (MD) was 0.53, where MD > 0.4 suggests that the network has a modular structure.

All genera in the network were assigned to bacteria phyla. Among these, Proteobacteria and Acidobacteria made up the two largest proportions, accounting for 38.78 and 20.41% of all nodes, respectively. When the distribution of nodes was modularized, all nodes were classified into three major modules (>10 nodes). Based on betweenness centrality scores, the top three genera identified were *Bryobacter*, GR-WP33-30, and *Rhizomicrobium*, which indicates the critical roles these microbes play as keystone taxa in the co-occurrence network.

Modules varied in their environmental associations, which indicates that taxa from the same module were ecologically similar (Supplementary Table S1). For instance, the abundance of genera in module I showed no significant correlations with any environmental factors, whereas the other two modules strongly correlated with such factors. In addition, the abundances of genera in the three major modules were negatively correlated with soil pH, nutrients, and water content, which suggests that these modules were composed primarily of oligotrophic, acidophilic, and drought-tolerant taxa.

## Discussion

### Responses of Soil Environmental Properties to the Vegetation Types

Both vegetation succession and afforestation can enhance soil organic C and N dynamics by increasing SOM input and decreasing the decomposition rate (Grünzweig et al., 2007; Morris et al., 2007; Zhu et al., 2012; Cheng and An, 2015; Nadal-Romero et al., 2016). Furthermore, vegetation restoration can increase the water-holding capacity of the soil (Selma, 2014; Zhang et al., 2016). In the present study, TN, SOC, and SM content generally increased as plant communities changed from grass to shrub and forest, which suggests that vegetation restoration accelerates organic matter accumulation and improves soil moisture conditions in Karst ecosystems. By contrast, a decreasing trend in pH was observed as vegetation restoration progressed, in accordance with previous studies (Robertson and Vitousek, 1981; Schipper et al., 2001; Dmowska and Ilieva-Makulec, 2006; Holtkamp et al., 2008; Zhao et al., 2014). Compared to *Quercus aquifolioides* × *Lithocarpus dealbatus* secondary forest, *Pinus armandii* pure plantation and *Pinus yunnanensis* × *Alnus ferdinandi-coburgii* mixed plantation exhibited lower pH values, which may have been due to acidification caused by coniferous afforestation (Brand et al., 1986; Jönsson et al., 2003). The native plants in Karst calcareous soil are mainly calcicoles, which accumulate amount of calcium in their leaves (White and Broadley, 2003). In the present study, a lower calcium accumulation capacity in these two calcifuge *Pinus* foliage may have reduced the calcium content of leaf litter and consequently by a decrease of pH value in topsoil. Besides, various origins of root-mediated changes such as organic acid may also affect soil pH (Hinsinger et al., 2003). Tree species was the most important factor determining the C:N ratio in European forest soils (Cools et al., 2014). In this study, the *Pinus armandii* plantation and *Pinus yunnanensis* × *Alnus ferdinandi-coburgii* mixed plantation had higher soil C:N ratios than other vegetation types, perhaps because of the divergent chemical characteristics of conifers compared to other plant species (Laganière et al., 2013; Hume et al., 2016). These results suggest that both vegetation restoration stage and chemical characteristics of the plant species present impact soil properties.

### Vegetation-Associated Edaphic Impacts on Bacterial Community Structure

The critical role of pH in shaping bacterial community structure is well characterized (Fierer and Jackson, 2006; Lauber et al., 2009; Chu et al., 2010; Griffiths et al., 2011; Ling et al., 2016). In the present study, soil pH was closely correlated with bacterial diversity across Karst revegetation sites. Moreover, soil microbial communities were influenced primarily by soil pH. The strong correlation between soil pH and microbial distribution could be due to the relatively narrow growth tolerances exhibited by most bacterial taxa. Indeed, each type of microorganism has an optimal pH value, and a slight change in pH might favor distinct bacterial taxa. Therefore, pH is a universal factor for predicting bacterial diversity and community structure (Fierer and Jackson, 2006; Singh et al., 2012). We observed a significant increase in the abundance of Acidobacteria with decreased pH, which has also been observed across terrestrial soil types (Eichorst et al., 2007). Calcium ion is implicated in a broad array of bacterial functions, including heat shock, pathogenicity, chemotaxis, differentiation, and the cell cycle (Norris et al., 1996). Ca^2+^ is closely related to bacterial community structure in typical Chinese forest soils (Xia et al., 2016). In this study, bacterial richness and diversity demonstrated significant positive correlations with Ca^2+^, which implies that the available calcium content acts as a determining factor in shaping soil bacterial populations and activity. In addition, soil moisture is an important factor driving microbial diversity across Karst vegetation types. Changes in soil water conditions affect oxygen content and substrate availability and consequently the microbial community (Yu et al., 2012; Banerjee et al., 2016). This finding is in agreement with previous studies in Antarctic soils (Yergeau et al., 2007; Chong et al., 2010), Canadian low Arctic tundra (Chu et al., 2011), the Yellow River Estuary in China (Yu et al., 2012), and Beilu River permafrost soils (Zhang et al., 2013).

In our study, soil bacterial diversity was negatively correlated with soil nutrient concentrations (SOC and TN). This pattern may indicate that low-resource environments in grassland and shrubbery lead to more unique niches, whereas relatively high-resource forestland habitats are less inclined to microbial niche differentiation, which has a major impact on microbial diversity (Bakker et al., 2013; Schlatter et al., 2015). Soil resource elemental stoichiometry plays an essential role in bacterial diversity and community composition (Högberg et al., 2007; Wan et al., 2015; Delgado-Baquerizo et al., 2017). In this study, significant relationships of C:N ratio with bacterial diversity and community structure were also observed, which indicates that variables associated with nitrogen transformations may be crucial determinants of bacterial community structure (Shi et al., 2015). Taken together, these results indicate that vegetation-associated soil properties play a vital role in determining bacterial community composition.

### Co-occurrence Patterns of Bacteria during Vegetation Restoration

The most abundant phyla studied in the co-occurrence network were Acidobacteria and Proteobacteria, which indicates that these generalists are adapted to a variety of environments (Jiao et al., 2016). Moreover, the CC value of 0.72 for the bacterial co-occurrence network in this Karst region was higher than those reported for other ecosystems (Ju et al., 2014; Peng et al., 2014), which demonstrates stronger correlations in this ecological network. The fact that *Bryobacter*, GR-WP33-30, and *Rhizomicrobium* had the top three betweenness centrality values indicates the importance of these nodes in the co-occurrence network. *Bryobacter*, an aerobic chemo-organotrophic bacterium that utilizes various sugars, polysaccharides, and organic acids, plays an important role in the biogeochemical carbon cycle (Dedysh et al., 2016). *Rhizomicrobium* is a symbiotic mycorrhizophere bacterium and is crucial for nitrogen fixation (Ueki et al., 2010). GR-WP33-30 has also been identified as a keystone species in previous research, and future work is needed to better understand the role of this bacterium in co-occurrence networks (Ma et al., 2016). Thus, all three keystone taxa may play critical roles in ecological processes.

Microbial species interactions are crucial to the structure and dynamics of soil bacterial communities (Czárán et al., 2002; Prosser et al., 2007; Hibbing et al., 2010; Schlatter et al., 2015). Correlation-based network analysis has been successfully used to explore the co-occurrence patterns of microbial communities (Barberán et al., 2012; Purkamo et al., 2015; Chao et al., 2016; Ma et al., 2016; Jiao et al., 2016; Pérez-Valera et al., 2017). Network analysis showed non-random co-occurrence patterns in microbial communities. Note that the 10 strongest positive correlations were all between different phyla, which indicates that metabolic cooperation may play an important role in shaping species co-occurrence (Zelezniak et al., 2015). Moreover, the different correlations between the abundance of genera and soil characteristics among modules indicate specific ecological characteristics in these assemblies. These non-random assembly patterns of bacteria indicate the dominance of species interactions and environmental filtering in shaping community structure.

## Conclusion

In the present study, we determined the effects of vegetation restoration and microbial interactions on the structure of bacterial communities in five vegetation types in KRD areas. Our work suggested that variations in soil physiochemical properties following revegetation led to shifts in the structure of the bacterial communities. Coniferous-broadleaved mixed plantation showed more similar bacterial community structure with secondary forest than coniferous plantation, indicating a better effect on ecological restoration of KRD land. Soil pH emerged as the major determinant of bacterial community characteristics. *Bryobacter*, GR-WP33-30, and *Rhizomicrobium* are the keystone taxa in KRD areas of southwestern China. Finally, we demonstrated non-random co-occurrence and modular patterns of bacterial communities. This information improves understanding of microbial responses to vegetation restoration in degraded karst regions.

## Author Contributions

In this study work, SL was responsible for experiment design and writing guidance, LX and HR were responsible for experiment performance and paper writing, XL and XY were responsible for experimental data processing and analysis.

## Conflict of Interest Statement

The authors declare that the research was conducted in the absence of any commercial or financial relationships that could be construed as a potential conflict of interest.

## Footnotes

1 http://drive5.com/uparse/

2 http://rdp.cme.msu.edu

3 http://bonsai.hgc.jp/mdehoon/software/cluster/index.html

4 http://sourceforge.net/projects/jtreeview.htm

## Supplementary Material

FIGURE S1 Rarefaction analysis for each sample.

FIGURE S2 The proportion of the bacterial phyla/subphyla under five vegetation types. The phylum Proteobacteria contains the four subphyla of Alphaproteobacteria, Betaproteobacteria, Deltaproteobacteria and Gammaproteobacteria. G, Grassland; S shrubbery; SF, secondary forest; MP, mixed plantation; PP, pure plantation.
