# Supplementary figures and images for: Soil Bacterial Community Structure and Co-occurrence Pattern during Vegetation Restoration in Karst Rocky Desertification Area
Source: Front Microbiol. 2017 Dec 1;8:2377. doi: 10.3389/fmicb.2017.02377 (PMC5717032; doi:10.3389/fmicb.2017.02377)

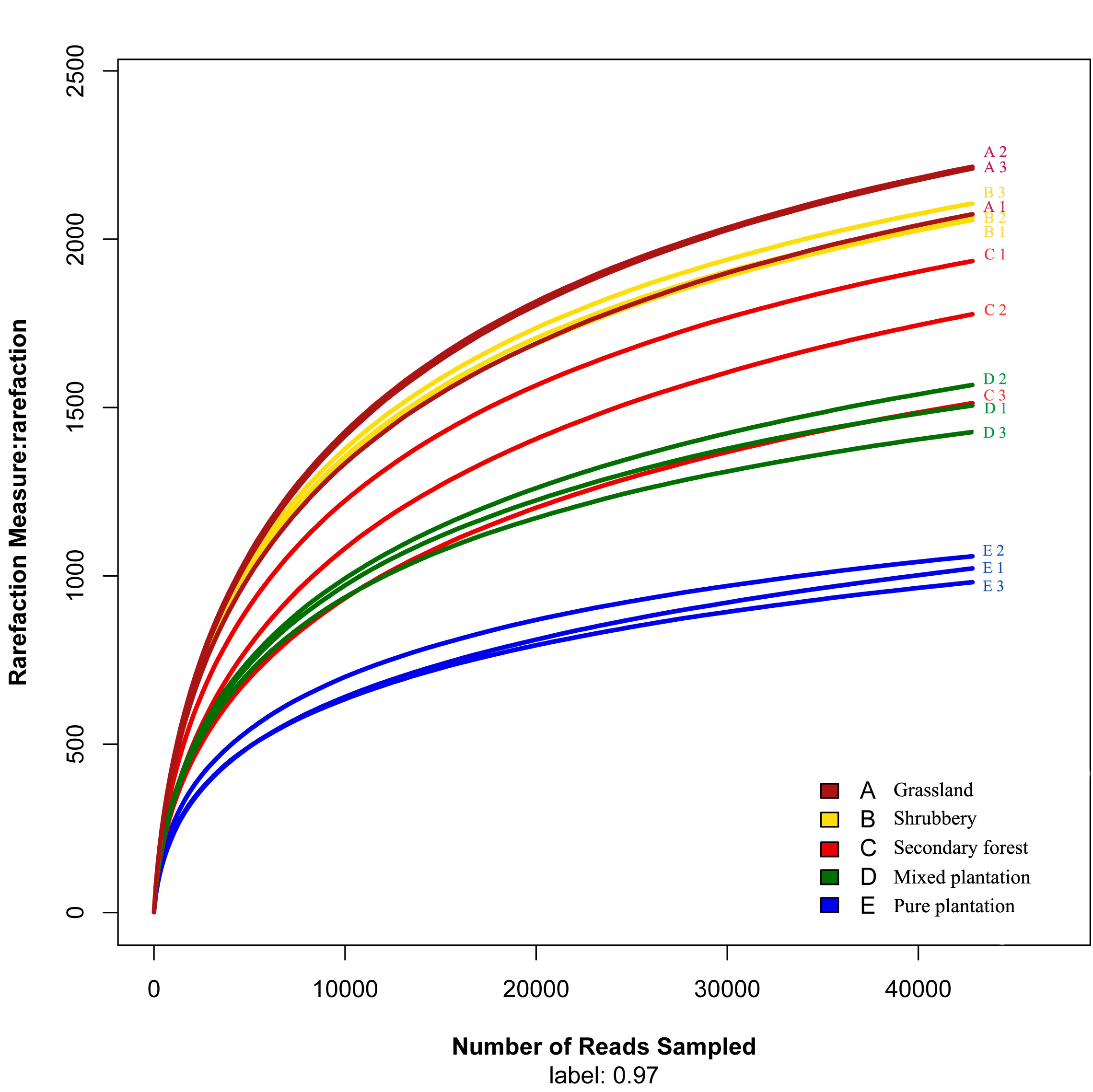

Supplement: FIGURE S1 — Rarefaction analysis for each sample. [file Image_1.JPEG]

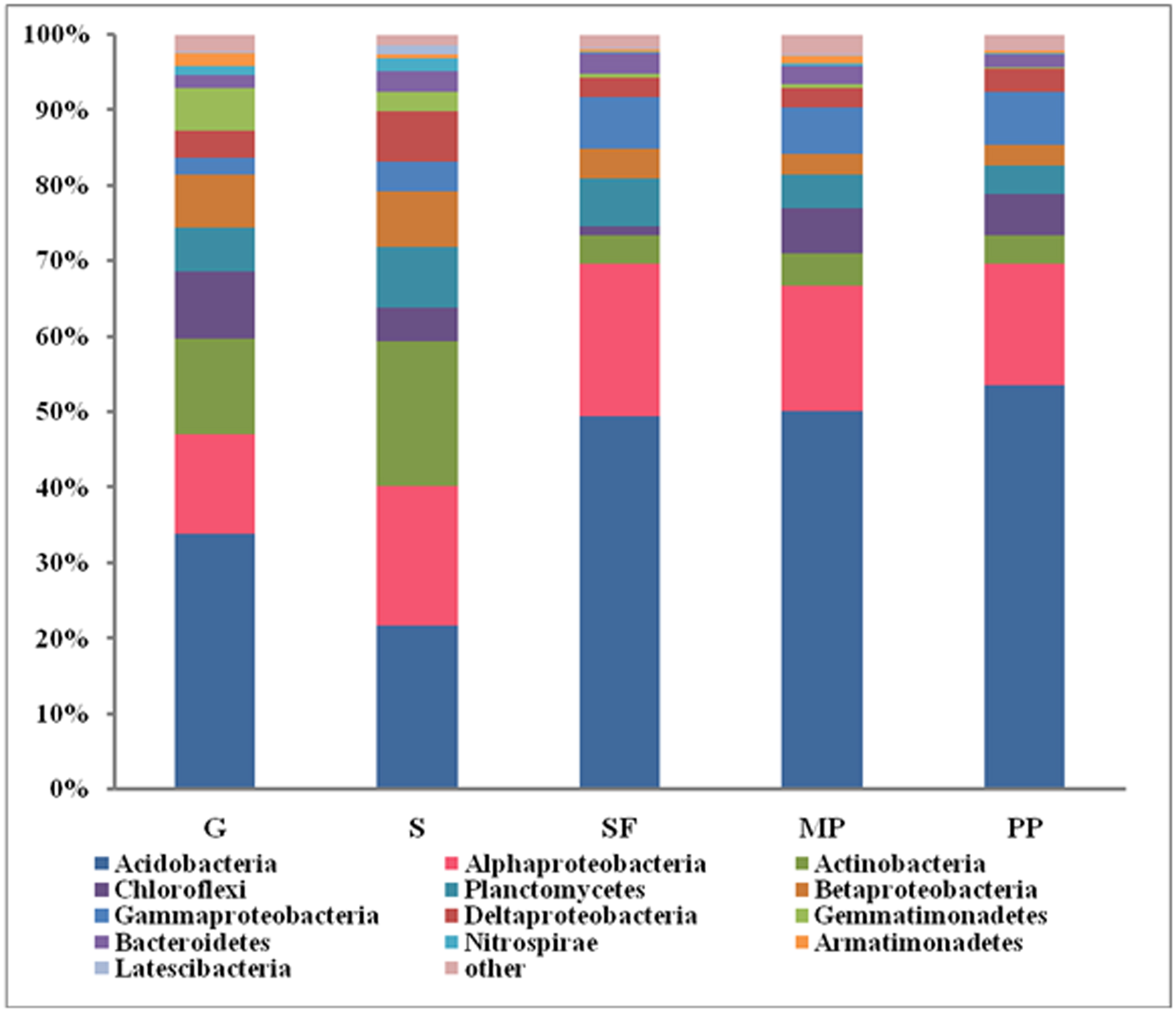

Supplement: FIGURE S2 — The proportion of the bacterial phyla/subphyla under five vegetation types. The phylum Proteobacteria contains the four subphyla of Alphaproteobacteria, Betaproteobacteria, Deltaproteobacteria and Gammaproteobacteria. G, Grassland; S shrubbery; SF, secondary forest; MP, mixed plantation; PP, pure plantation. [file Image_2.JPEG]
