# Supplementary material for: Soil Bacterial Community Structure and Co-occurrence Pattern during Vegetation Restoration in Karst Rocky Desertification Area
Source: Front Microbiol. 2017 Dec 1;8:2377. doi: 10.3389/fmicb.2017.02377 (PMC5717032; doi:10.3389/fmicb.2017.02377)
Supplement: Supplementary file 3 [file Table_1.DOCX]

**Table S1** Correlation between Physicochemical factor and modules of co-occurring taxa.

| Physicochemical and biological factors | Modules | | |
| --- | --- | --- | --- |
|  | Module 1 | Module 2 | Module 3 |
| pH | -0.224 | **-0.847** | **-0.861** |
| Ca | -0.147 | **-0.673** | **-0.833** |
| SM | -0.197 | **-0.855** | **-0.787** |
| SOC | -0.315 | **-0.810** | **-0.688** |
| C/N | -0.488 | **-0.733** | **-0.525** |
| TN | -0.482 | **-0.698** | **-0.754** |

Significant correlation coefficients are noted as bold text where P < 0.05
