# Supplementary material for: Soil Bacterial Community Structure and Co-occurrence Pattern during Vegetation Restoration in Karst Rocky Desertification Area
Source: Front Microbiol. 2017 Dec 1;8:2377. doi: 10.3389/fmicb.2017.02377 (PMC5717032; doi:10.3389/fmicb.2017.02377)
Supplement: Supplementary file 4 [file Table_2.DOCX]

**Tab. S2** Taxonomic affiliation of top 50 OTUs among five vegetation types

| **OUT number** | **Taxonomic affiliation** |
| --- | --- |
| OTU96 | d__Bacteria; p__Acidobacteria; c__Acidobacteria; o__Acidobacteriales; f__Acidobacteriaceae_Subgroup_1; g__uncultured; s__uncultured_Acidobacteriaceae_bacterium |
| OTU147 | d__Bacteria; p__Acidobacteria; c__Acidobacteria; o__Subgroup_3; f__Unknown_Family; g__Bryobacter; s__uncultured_Acidobacteria_bacterium |
| OTU152 | d__Bacteria; p__Proteobacteria; c__Betaproteobacteria; o__Burkholderiales; f__Comamonadaceae; g__Piscinibacter; s__uncultured_bacterium |
| OTU338 | d__Bacteria; p__Acidobacteria; c__Acidobacteria; o__Acidobacteriales; f__Acidobacteriaceae_Subgroup_1; g__Telmatobacter; s__uncultured_bacterium |
| OTU386 | d__Bacteria; p__Acidobacteria; c__Acidobacteria; o__Subgroup_2; f__norank; g__norank; s__uncultured_bacterium |
| OTU447 | d__Bacteria; p__Acidobacteria; c__Acidobacteria; o__Subgroup_2; f__norank; g__norank |
| OTU678 | d__Bacteria; p__Proteobacteria; c__Betaproteobacteria; o__Burkholderiales; f__Burkholderiaceae; g__Burkholderia |
| OTU686 | d__Bacteria; p__Proteobacteria; c__Gammaproteobacteria; o__Xanthomonadales; f__Xanthomonadales_Incertae_Sedis; g__Acidibacter; s__uncultured_Steroidobacter_sp. |
| OTU892 | d__Bacteria; p__Proteobacteria; c__Alphaproteobacteria; o__Rhizobiales |
| OTU1191 | d__Bacteria; p__Acidobacteria; c__Acidobacteria; o__Acidobacteriales; f__Acidobacteriaceae_Subgroup_1; g__uncultured; s__uncultured_bacterium |
| OTU1276 | d__Bacteria; p__Acidobacteria; c__Acidobacteria; o__Subgroup_6; f__norank; g__norank; s__uncultured_Holophaga_sp. |
| OTU1298 | d__Bacteria; p__Acidobacteria; c__Acidobacteria; o__Acidobacteriales; f__Acidobacteriaceae_Subgroup_1; g__uncultured |
| OTU1358 | d__Bacteria; p__Acidobacteria; c__Acidobacteria; o__Acidobacteriales; f__Acidobacteriaceae_Subgroup_1; g__Acidobacterium; s__uncultured_Acidobacteria_bacterium |
| OTU1369 | d__Bacteria; p__Acidobacteria; c__Acidobacteria; o__Subgroup_2; f__norank; g__norank; s__uncultured_Acidobacteria_bacterium |
| OTU1536 | d__Bacteria; p__Acidobacteria; c__Acidobacteria; o__Acidobacteriales; f__Acidobacteriaceae_Subgroup_1; g__uncultured; s__uncultured_forest_soil_bacterium |
| OTU1666 | d__Bacteria; p__Acidobacteria; c__Acidobacteria; o__Subgroup_3; f__Unknown_Family; g__Candidatus_Solibacter |
| OTU1707 | d__Bacteria; p__Proteobacteria; c__Alphaproteobacteria; o__Rhizobiales; f__Bradyrhizobiaceae; g__Bradyrhizobium |
| OTU1750 | d__Bacteria; p__Acidobacteria; c__Acidobacteria; o__Subgroup_2; f__norank; g__norank; s__uncultured_Holophaga_sp. |

**Tab S2, Continued**

| **OUT number** | **Taxonomic affiliation** |
| --- | --- |
| OTU1764 | d__Bacteria; p__Acidobacteria; c__Acidobacteria; o__Subgroup_2; f__norank; g__norank; s__uncultured_bacterium |
| OTU1799 | d__Bacteria; p__Acidobacteria; c__Acidobacteria; o__Subgroup_6; f__norank; g__norank |
| OTU1825 | d__Bacteria; p__Proteobacteria; c__Alphaproteobacteria; o__Rhizobiales; f__Xanthobacteraceae; g__Variibacter; s__uncultured_forest_soil_bacterium |
| OTU1980 | d__Bacteria; p__Acidobacteria; c__Acidobacteria; o__Subgroup_7; f__norank; g__norank; s__uncultured_bacterium |
| OTU2006 | d__Bacteria; p__Proteobacteria; c__Alphaproteobacteria; o__Rhodospirillales; f__DA111; g__norank |
| OTU2325 | d__Bacteria; p__Proteobacteria; c__Betaproteobacteria; o__Burkholderiales; f__Burkholderiaceae; g__Burkholderia |
| OTU2328 | d__Bacteria; p__Acidobacteria; c__Acidobacteria; o__Subgroup_2; f__norank; g__norank; s__uncultured_Acidobacteria_bacterium |
| OTU2342 | d__Bacteria; p__Acidobacteria; c__Acidobacteria; o__Subgroup_3; f__Unknown_Family; g__Bryobacter; s__uncultured_Holophaga_sp. |
| OTU2375 | d__Bacteria; p__Proteobacteria; c__Alphaproteobacteria; o__Rhizobiales; f__Xanthobacteraceae; g__uncultured; s__uncultured_Bradyrhizobium_sp. |
| OTU2458 | d__Bacteria; p__Actinobacteria; c__Actinobacteria; o__Gaiellales; f__uncultured; g__uncultured; s__uncultured_Rubrobacteridae_bacterium |
| OTU2534 | d__Bacteria; p__Acidobacteria; c__Acidobacteria; o__Acidobacteriales; f__Acidobacteriaceae_Subgroup_1 |
| OTU2557 | d__Bacteria; p__Acidobacteria; c__Acidobacteria; o__Subgroup_3; f__Unknown_Family; g__Bryobacter; s__uncultured_Acidobacteria_bacterium |
| OTU2579 | d__Bacteria;p__Acidobacteria; c__Acidobacteria; o__Acidobacteriales; f__Acidobacteriaceae_Subgroup_1; g__uncultured; s__uncultured_Candidatus_Koribacter_sp. |
| OTU2824 | d__Bacteria; p__Acidobacteria; c__Acidobacteria; o__Acidobacteriales; f__Acidobacteriaceae_Subgroup_1; g__uncultured |
| OTU2916 | d__Bacteria; p__Proteobacteria; c__Betaproteobacteria; o__Burkholderiales; f__Comamonadaceae |
| OTU2946 | d__Bacteria; p__Acidobacteria; c__Acidobacteria; o__Subgroup_3; f__Unknown_Family |
| OTU2964 | d__Bacteria; p__Acidobacteria; c__Acidobacteria; o__Subgroup_4; f__RB41; g__norank; s__uncultured_Acidobacteriaceae_bacterium |
| OTU3020 | d__Bacteria; p__Proteobacteria; c__Betaproteobacteria; o__Nitrosomonadales; f__Nitrosomonadaceae; g__uncultured |
| OTU3068 | d__Bacteria; p__Actinobacteria; c__Actinobacteria; o__Solirubrobacterales; f__Solirubrobacteraceae; g__Solirubrobacter |
| OTU3113 | d__Bacteria; p__Acidobacteria; c__Acidobacteria; o__Subgroup_2; f__norank; g__norank; s__uncultured_eubacterium_WD261 |
| OTU3223 | d__Bacteria; p__Proteobacteria; c__Deltaproteobacteria; o__GR-WP33-30; f__norank; g__norank; s__uncultured_Firmicutes_bacterium |
| OTU3231 | d__Bacteria; p__Proteobacteria; c__Gammaproteobacteria; o__Xanthomonadales; f__uncultured; g__uncultured; s__uncultured_eubacterium_WD260 |

**Tab S2, Continued**

| **OUT number** | **Taxonomic affiliation** |
| --- | --- |
| OTU3250 | d__Bacteria; p__Gemmatimonadetes; c__Gemmatimonadetes; o__Gemmatimonadales; f__Gemmatimonadaceae; g__uncultured; s__uncultured_Gemmatimonadetes_bacterium |
| OTU3278 | d__Bacteria; p__Acidobacteria; c__Acidobacteria; o__Acidobacteriales; f__Acidobacteriaceae_Subgroup_1; g__uncultured |
| OTU3329 | d__Bacteria; p__Acidobacteria; c__Acidobacteria; o__Subgroup_2; f__norank; g__norank; s__bacterium_Ellin7505 |
| OTU3346 | d__Bacteria; p__Acidobacteria; c__Acidobacteria; o__Acidobacteriales; f__Acidobacteriaceae_Subgroup_1; g__Candidatus_Koribacter |
| OTU3371 | d__Bacteria; p__Chloroflexi; c__JG37-AG-4; o__norank; f__norank; g__norank |
| OTU3472 | d__Bacteria; p__Proteobacteria; c__Alphaproteobacteria; o__Rhodospirillales; f__Rhodospirillales_Incertae_Sedis; g__Reyranella |
| OTU3502 | d__Bacteria; p__Acidobacteria; c__Acidobacteria; o__Acidobacteriales; f__Acidobacteriaceae_Subgroup_1; g__Granulicella |
| OTU3544 | d__Bacteria; p__Proteobacteria; c__Deltaproteobacteria; o__Desulfobacterales; f__Nitrospinaceae; g__Candidatus_Entotheonella; s__uncultured_bacterium |
| OTU3565 | d__Bacteria; p__Acidobacteria; c__Acidobacteria; o__Subgroup_2; f__norank; g__norank |
| OTU3626 | d__Bacteria; p__Acidobacteria; c__Acidobacteria; o__Subgroup_2; f__norank; g__norank |

d, kingdom; p, phylum; c, class; o, order; f, family; g, genus; s, species
